# Nurses’ perceptions regarding their own professionalism attributes to quality neonatal, infant and under-5 childcare

**DOI:** 10.1186/s12912-024-02375-0

**Published:** 2024-10-08

**Authors:** Dibolelo Adeline Lesao, Tinda Rabie, Welma Lubbe, Suegnet Scholtz

**Affiliations:** 1 School of Nursing Science, NuMIQ Focus Area, North-West University, Potchefstroom, South Africa; 2 North-West University, NuMIQ Focus Area, Vanderbijlpark, South Africa

**Keywords:** Attributes, Infants, Neonates, Under-5, Professional nurses, Professionalism, Quality of care

## Abstract

**Background:**

Professional nurses are trained to provide quality care. Despite the professional nurses’ acquired skill and professionalism attributes, the neonate, infant, and under-5-child mortality rates are high in sub-Saharan Africa. This health care report indicates that sub-Saharan Africa countries face a challenge in reaching the Sustainable Development Goal number 3 by the year 2030 (that is, ensuring healthy lives and reducing the mortality rates of children under 5). It has been reported that professionalism in nursing can improve the quality of care and positively change the health outcomes.

**Methods:**

This study employed a qualitative exploratory, descriptive design to explore and describe professional nurses’ own professionalism attributes to provide quality care to neonates, infants, and under-5 children in the North West province in South Africa. Eight naïve sketches of an all-inclusive sample of invited professional nurses (*N* = 25; *n* = 8) were received. The naïve sketch questions were based on the Registered Nurses’ Association of Ontario’s professionalism attributes. Tesch’s eight steps of data-analysis were used, with an independent coder’s assistance.

**Results:**

The categories included (1) knowledge, (2) spirit of inquiry, (3) accountability, (4) autonomy, (5) advocacy, (6) collegiality and collaboration, (7) ethics and values, and (8) professional reputation, and each category generated sub-themes.

**Conclusion:**

Professional nurses are aware of their own professionalism attributes in quality of care of neonates, infants and under-5 children; the ‘innovation and visionary’ attribute did not emerge, which should receive more attention to strengthen quality care. However, a new attribute, ‘professional reputation’, reflecting a South African culture-orientated attribute, emerged from the data collected.

**Supplementary Information:**

The online version contains supplementary material available at 10.1186/s12912-024-02375-0.

## Background

Nursing professionalism is a characteristic acquired through the education and career development of professional nurses (PNs). While undergoing education and training, the PNs are prepared to become competent, ethical and skilled practitioners, who will be capable of providing quality care [1]. The PNs independently assess, diagnose, manage and refer neonates, infants and children under-5 years old, in primary health care settings or in a multi-disciplinary team (MDT) in a hospital, guided by their policies, protocols and the professionalism attributes [2, 3]. It has been established in literature [4, 5] that nursing professionalism directly affects quality-care provision to patients, and neonates, infants and children under 5. In 2016, the United Nations introduced Sustainable Development Goals (SDG) 2030, with SDG 3.2 focusing on an improved health outcome for the neonate, infant and under-5 child health. Globally, neonates and children under-5 die from preventable diseases such as diarrhoea, respiratory infections, measles and malaria [6, 7]. The SDG 3.2 global target is to reduce these preventable deaths of neonates to 12 per 1000 live births, and to 25 per 1000 live births for children under-5, by the year 2030 [8]. However, the trends seen in other studies [7–9] show that only 68% of countries will reach the neonatal mortality rate, while the desired under-5 mortality rate will be reached by 75% of countries. It is concerning that, despite professionalism attributes acquired by PNs, sub-Saharan Africa countries are among the ones that will not reach the SDG 3.2 targets [8–10].

The North West province in South Africa (SA), where this study was conducted, contributed 3171 of 31938 deaths of neonates, infants and children under-5 combined in the country [11]. It is therefore evident that the Integrated Management of Childhood Illnesses (IMCI), the Expanded Programme on Immunisation (EPI) and nutritional guidelines alone, are not sufficient for the PNs to reduce the neonatal, infant and under-5 mortality rates in SA [12]. This inspired this study, which explored and described PNs’ professionalism attributes to provide quality of care [13, 14].

As is evident in the literature, the nursing professionalism concept has various definitions [4]. The Registered Nurses’ Association of Ontario’s (RNAO’s) 2007 best practice guidelines clearly describe the eight attributes of nursing professionalism. The RNAO’s [15] eight attributes of nursing professionalism are namely 1) knowledge, 2) spirit of inquiry, 3) accountability, 4) autonomy, 5) advocacy, 6) innovation and visionary, 7) collegiality and collaboration, and 8) ethics and values. However, in more recent years, authors [4] have defined nursing professionalism as “multidimensional and evidenced by the knowledge, attitudes, and behaviours that underlie successful clinical practice”, with multidimensional, dynamic and culture-orientated attributes [4]. Additionally, the International Society for Professional Identity [16] highlighted that knowledge, accountability, ethics and values were at the root of nursing identity and form part of professionalism. These attributes are aligned with the South African Nursing Council (SANC) Code of Ethics [17], stating that nursing professionalism should demonstrate accountability, patient autonomy, ethical practice, quality of care, and professional reputation; improper, disgraceful, dishonourable and unworthy actions are regarded as unprofessional conduct [1]. Nursing professionalism evolves [18], and definitions by several authors [4, 16] are recognised; however, this study was conceptualised from the RNAO nursing professionalism attributes as a framework, as they are well aligned with the South African context and guidelines on nursing-care provision.

Nursing professionalism has been studied, as a concept, in adult clinical-nursing care as well as student settings; however, a limited number of studies are found in the context of neonatal, infant and under-5 childcare, particularly in low- and middle-income countries like SA. This study’s focus, the perceptions of PNs’ professionalism attributes to quality care in the context of neonates, infants and children under-5 in South Africa, have not been previously explored. Achieving SDG 3 in low- and middle-income countries such as SA is a steep climb, considering the disease burden [19, 20]. Neonates, infants and children under 5 are the most affected, and fast progress towards quality health care to ensure healthy lives and promote wellbeing is essential [8, 21]. Therefore, exploring the identified gap of PNs’ perceptions of their own nursing professionalism attributes in the care of neonates, infants and children under 5, in North West province and in SA, can contribute to quality care and consequently contribute to a positive outcome for SDG 3.2.

## Methods

### Aim

This study aimed to explore and describe the professionalism attributes of PNs to provide quality care to neonates, infants, and children under-5 in North West province.

### Design

The study used a qualitative, exploratory, descriptive design [22].

### Sample

To achieve the SDG in the sub-Sahara Africa context, it is vital to bring in more stakeholders to reduce the health care challenges [21]. As an example, the Starfish Project is a capacity-building collaboration between the North West Department of Health (NWDoH) and North-West University, aimed at the PNs working in Primary Health Care (PHC) and hospital settings with neonate, infant and under-5 child populations. Starfish Project provides refresher training to update the PNs’ skills and supports child-care programmes that are already in place.

An all-inclusive sample of (*N* = 25) PNs enrolled in the Starfish Project; (*n* = 8) participated in the study, resulting in a 32% response rate. The PNs were included if they were involved in the Starfish Project; had been licensed with the SANC for at least one year; specifically worked with neonates, infants and children under-5; were literate in English; had a WhatsApp-enabled phone; and were willing to participate.

### Data collection

Data were collected from October 2020 to May 2021, during the Covid-19 pandemic, via the WhatsApp platform.

Naïve sketches were used for data collection, to enable the PNs to reflect on their nursing professionalism attributes in their work day, and enable them to write down their perceptions in their own space and convenient time. The researcher identified three experts in the field of nursing neonatal, infant and the under 5 child, to check the naïve-sketch booklet to determine if the questions were well understood before data collection commenced. No changes were required, from the feedback received.

The data-collection tool consisted of two sections. Section A contained questions on demographic data; marital status, age, gender, number of children, home language, nursing qualification degree/diploma and years of work experience. Section B consisted of a fictional letter in which the PNs were requested to reply to the naïve sketch questions. The questions were based on the RNAO [15] nursing professionalism attributes framework. Two or more attributes were clustered and renamed to form an open-ended question, to trigger the PN into responding to the questions posed in Table 1.

**Table 1:** Demographic questions and interview schedule

Section A: Demographic data													
**Age**:	18–29	30–35		36–40	41–45	46–50	51–55				56–60	60+	
**Gender**:	Male	Female											
**Marital status**:	Single	Married	Widowed		Divorced	In a relationship			Other				
**Number of children**:	1	2	3		4	5+							
**Home language**:													
**Qualification**:	Degree	Diploma											
**Working experience (years)**:	1–5	6–10	11–15		16–20	21–25		26–30		30+			
**Section B: Naïve sketch**													
Question 1 What is your perception of nursing professionalism concerning interpersonal aspects, responsibility towards professional development, image, collegial relationships, the physical environment of your facility and any other relevant aspect of nursing professionalism that I, Joy, did not mention?													
1.1 My perceptions of how my interpersonal relationships contribute to quality neonatal, infant and under-5 child care include the following……………………………………………………….													
1.2 My perceptions of how my responsibility for professional development contributes to quality neonatal, infant and under-5 child care include the following…………….……….…….…….													
1.3 My perceptions of how my professional image contributes to quality neonatal, infant and under-5 child care include the following……………………………….…………………………													
1.4 My perceptions of how my collegial relationships contribute to quality neonatal, infant and under-5 child care include the following…………………………………………………………													
1.5 My perceptions of how the physical environment of the facility contributes to quality neonatal, infant and under-5 child care include the following……………………………………………………													
1.6 The following are my perceptions regarding other relevant aspects about the professionalism that contribute to quality neonatal, infant and under-5 child care………………………………… …………………………………………………………………………………………………….													
Question 2 2.1 In my own perception from previous and current experience, PNs in the PHC setting need to maintain professionalism because…………………………………………………….………													

A photo of both sections was sent to the participants via WhatsApp, which is encrypted and therefore secure, with the request that they answer each section and question separately on paper. The PNs wrote the naïve sketches at their own pace and in their own time when they felt comfortable. The PNs privately sent photos of their response to the first author’s private cellphone (mobile) number. Participants had at least four weeks to complete the data-collection process. Two reminder messages were sent via Short Message Services to encourage and remind PNs to continue writing if they were comfortable doing so. After the first author received the participants’ response photos, they were downloaded and saved on a password-protected computer in a folder named with a number, in order to minimise confusion during analysis. Each photo was then deleted from the first author’s cellphone.

### Ethical considerations

Ethical approval was granted by the Health Research Ethics Committee (HREC) of the North West University (NWU-00438-19-A1), followed by Policy, Planning, Research Monitoring and Evaluation from the NWDoH. The Starfish Project coordinator granted goodwill permission. A PowerPoint presentation of the study was made, and consent was explained by the researcher prior to the Covid-19 lockdown restrictions. An independent person distributed anonymised consent forms in separate envelopes to interested PNs, to take home to read and decide about participation. They were asked to return the unsigned forms at their next class within in a month. As the Covid-19 pandemic lockdown restricted movement before the return of consent forms, permission was granted by HREC to obtain informed consent telephonically without an independent person. The process involved both parties signing the informed consent form with their witnesses and sharing the signed pages via WhatsApp. The informed consent hardcopies were collected when lockdown levels were eased, and were locked in a cupboard for confidentiality.

### Data analysis

The demographic data in Section A were entered into an Excel spreadsheet, and data obtained in Section B were analysed using Tesch’s steps of content analysis [23] with the assistance of a co-coder. Two copies were printed from the downloaded photos of the naïve sketches, one each for the researcher and co-coder. First, the naïve-sketch photos were read carefully to obtain a sense of the whole data, and ideas were noted. Secondly, one naïve sketch was picked randomly and scrutinised without thinking about the details of the information, but rather to determine the underlying meaning. Thirdly, a list of topics was compiled, and similar topics were clustered together after several naïve sketches had been scrutinised. Topics were formed into columns named major, unique and/or leftover.

Fourthly, the list was inputted as data to abbreviate the topics and write codes next to the appropriate segments of the texts. This organising assisted in the emergence of new categories and codes. Fifthly, topics were categorised using descriptive words and reduced in number by grouping related topics. Interrelationships of categories were indicated by inserting a line between categories. Sixthly, abbreviations were finalised for each category and organised in alphabetical order. Seventhly, a preliminary analysis was done by grouping similar data together per category. Lastly, the researcher and co-coder held a discussion to reach consensus on which RNAO [15] nursing-attributes themes emerged. The researcher and co-coder reached consensus with only minor changes.

### Trustworthiness

Trustworthiness was demonstrated according to the standards of trustworthiness set out by Lincoln [24]. To ensure credibility of the study, the researcher immersed herself in the research process. The data collected and analysed reflected only the participants’ perceptions, without bias from the researcher. Data saturation was reached, and the coding process was done by an independent co-coder. Transferability strategy involved a detailed research design, and the methodology process is clearly described. The collected data used to reach a finding emerged from knowledgeable PNs caring for the neonate, infant and the under-5 child. The researcher was consistent and precisely applied the described research design and methodology throughout the research process, in order to enhance dependability. To ensure confirmability, only data provided by the participants were used. An independent co-coder was involved in data analysis to ensure non-bias, assisted by supervisors as experts in the field of research. The researcher conducted a thorough literature review to support the findings (see Table  S1).

## Results

### The participants

All eight participants were female. Half of the participants were aged between 18 to 29 years and the older participants were between 36 to 40 years. There was an equal number of three single and three married participants; the remaining two were in a relationship. Most of the participants had one child, with one participant having three children. The dominant language was Setswana (see Table S2). A diploma in nursing was the highest qualification for five participants, and the remaining three held a university degree. Work experience ranged from 1 to 5 years for five participants (see Table S2).

### Findings

Eight categories of themes, with sub-themes, were yielded by the study, namely, (1) knowledge, (2) spirit of inquiry, (3) accountability, (4) autonomy, (5) advocacy, (6) collegiality and collaboration, (7) ethics and values, and (8) professional reputation.

Quotations from the interviews are provided as examples of common themes. The coding at the end of each excerpt follows a formula; for example in the code ‘(1/8/F/36–40)’, 1/8 identifies the naïve sketch number, F refers to female, and 36–40 refers to the participant’s age group. Table S3 (below) describes the perceptions of PNs regarding their own professionalism attributes to provide quality care to neonates, infants and children under 5, and includes sub-themes and the PNs “verbatim” responses/quotes.

The following section outlines the findings presented in Table S3. There are eight categories with themes and sub-themes from the PNs’ responses.

### Theme 1: knowledge

‘Knowledge’ referred to knowledge-acquisition as a theme. The PNs’ approach to knowledge-acquisition involved the sub-themes in-service training, new guidelines, and evidence-based practice. In-service training enables the PNs to give feedback and share knowledge and skills with colleagues who could not attend courses and training, or be updated on new health guidelines, policies, or programmes.

### Theme 2: spirit of inquiry

The spirit of inquiry was exhibited through the theme lifelong learning, which included the sub-themes short courses and advanced courses, diplomas and degrees. The PNs are aware of the continuous health care changes that require lifelong learning. PNs are not solely reliant on the Department of Health (DoH) for training. They are taking responsibility for their professional standards by enrolling or registering themselves in advanced health care education courses, diplomas and degrees with various nursing education institutions to improve competency and attain specialised qualifications in order to provide quality care to neonates, infants and children under-5.

### Theme 3: accountability

Accountability included the theme of responsibility for good practice, which included the sub-theme of the scope of practice, acts and omissions, and mismanagement. The PNs practise firstly according to the set regulations and scope of practice for the profession set by the SANC. At the workplace, more policies and guidelines are available to be used by nurses to avoid malpractice and ensure safe practice.

### Theme 4: autonomy

Autonomy included enhancing career independence by empowering the PNs through campaigns, seminars and courses for PNs which were treated as sub-themes. There is a need for the PNs to be allowed to be part of the decision-making process in the health care system, as steps towards improving practice.

### Theme 5: advocacy

Advocacy included the theme of communication between the MDTs, with record-keeping and referral as the sub-themes. Record-keeping during and before referrals is important to ensure continuity of care.

### Theme 6: collegiality and collaboration

The collegiality and collaboration theme included collegial interrelationships, with the sub-themes of mutual respect and trust, good collegial communication skills, teamwork, and team spirit. Mutual respect and trust amongst colleagues include consideration and sensitivity towards each other’s cultural backgrounds, and acknowledging the MDT’s input for the care and management of the child. A positive working environment is created through trust and respect and facilitates the goal of providing quality care. Quality care for neonates, infants and children under five years is an MDT effort. Verbal and non-verbal communication ensures continuity of care. Poor teamwork or team spirit in the MDT causes dysfunction and discord in health care delivery, jeopardising child health care. The PNs highlighted the need for cleaners, mentor mothers, social workers, doctors, health promoters and dietitians to adopt one vision for providing quality care.

### Theme 7: ethics and values

The ethics and values theme included sub-themes of forming trusting and respectful relationships, confidentiality and privacy, good communication skills and health education, as components of the PNs’ perception of ethical practice. Trust and respect for the parents/caregivers increases their confidence to care for the unwell child and helps to ensure they attend health care appointments. For the PNs to be trusted, they need to uphold the principles of privacy and confidentiality. PNs need to be welcoming, flexible, and active listeners to reassure the parents/caregivers that, at any stage of their child’s illness, they can seek assistance before complications occur. A good listener and friendly PN creates a safe space for parents/caregivers to give an accurate health history and to make informed health decisions for their child. Health education ensures continuity of care at home. It requires a good relationship between the nurse and parent/caregiver so that the receiver, the parent/caregiver, can accept, process and apply the health advice given, for the further care of their child, at home or in the community.

### Theme 8: professional reputation

The professional reputation theme refers to the reputation of both the PN and the healthcare facility. Regarding the PN, the sub-themes of clean uniforms, distinguishing devices, and name tags – as prescribed by the nursing regulations – and personal hygiene (clean hair and nails) emerged. Good appearance is judged on the cleanliness of the hair, which must not get in the way when the PN is performing duties. Long nails are not acceptable because of the possibility of cross-infection or causing cuts on the baby’s skin. Dirty uniforms can transmit infections to the child, which is unprofessional. Shoes must be safe for the workplace, to withstand slippery floors and long hours of work, so that more children can be attended to. Self-care creates a good impression that shows commitment to the profession. An acceptable nurse reputation (image) eases the parent/caregiver’s mind, and they trust that a skilled, professional person is treating their child.

The health care facility theme included the sub-themes infection control, safety and needle- disposal bins; adequate equipment, medication and nursing staff; and an available, pleasant atmosphere in consultation rooms. PNs’ perceptions are that neonates, infants, and children under 5 are vulnerable. Proper infection-control measures such as sanitation, and clean and organised work areas, are vital. Poor sanitation exposes the child to further infections. Children must be kept safe from harm, and a disorganised PN creates the opportunity for injuries in the facility. A safe facility means floors must be chip-free, clean, dry, and free of objects that may cause falls. It must have suitable waste-disposal bins for waste segregation. Resource availability comprises equipment, medication, and nursing staff. To deliver quality care for neonates, infants and children under-5, the correct and working equipment, medication and nursing staff are required – this was more so during the Covid-19 pandemic. PNs mentioned that consulting rooms should be available and have a pleasant atmosphere. Rooms should have colourful walls, animal pictures, and toys to create a pleasant atmosphere in the clinic for neonates, infants and children under 5.

## Discussion

In this study, perceptions of PNs regarding their own nursing professionalism attributes to provide quality care to neonatal, infant and children under 5 were explored and described, and eight themes emerged.

The study’s results are consistent with the findings of previous studies – that knowledge-acquisition is a deliberate and evolving process to achieve better health care outcomes for neonates, infants and children under 5 [25]. Often, PNs felt that clinical guidelines did not always meet the needs of the health care practice; therefore, they need to be part of the planning and implementation process to achieve successful health outcomes [26, 27]. The benefit of specialised care – achieved through life-long learning and advanced training, such as an advanced diploma or degree and continuous personal development – can improve health outcomes for neonates, infants and children under-5 [4, 28, 29].

Accountability ensures patient safety. The PNs in this study value the scope of practice to guide them in their nursing care; however, in some countries, such as Ireland and America, PNs experienced limitations and conflicting roles when adhering to their scope of practice [30, 31]. Registration with a professional body is another way for the PN to show accountability; it allows the statutory body to intervene in acts and omissions committed in clinical practice [1, 32]. Accountability can be promoted through effective leadership that enforces positive work relations and reporting of unethical practices [33, 34].

The PNs also felt that autonomy was not valued and was not visible in health policymaking. The PNs believe that public awareness campaigns, seminars, workshops with experts, and inclusion in planning and policymaking will strengthen their decision-making skills, as also emphasised by [35]. The role of nurse advocacy involves the parent/caregiver, the MDT, and the organisational regulations and laws [36, 37]. The PNs acknowledged that nurse advocacy does not entail taking away the decision-making role of the parent/caregiver. Advocacy is mediating and empowering parents/caregivers with knowledge, to enable them to exercise informed decision-making for their child’s health [38, 39].

Collegiality and collaboration facilitate coordinated and quality care. A trusting relationship is characterised by the freedom of team members to perform their duties without judgement on their limitations [40, 41]. Teamwork and team spirit is built on communication, and disagreements must be resolved promptly. A patient who observes good teamwork among the staff trusts the team, and care is delivered faster and better [42]. Parents/caregivers feel they can share important health information regarding the child’s illness or their social-wellbeing challenges when the PN is welcoming [43]. The PNs disapproved of sharing children’s health information with anyone not directly involved with their care, believing it undermines professionalism and may expose the child to harm [44]. This finding correlates with previous studies on the awareness and importance of the child’s privacy and confidentiality in their field of childcare [45, 46].

Ethical behaviour, compassion, and competence describe professionalism for PNs [47, 48]. Showing respect when communicating with parents/caregivers builds trust. The caregivers/parents may not be open to information because their child is sick. Health education must be tailored and delivered with consideration to the existing circumstances and the needs of the child and parents/caregivers [39, 49].

The PN from the MDT is the first contact person the parent/caregiver meets at the health care facility. At the hospital, they are caring for the sick child 24 h a day, and a bad attitude experienced by the parent/caregiver from the PN can taint their relationship [37, 50]. Nurses’ professional reputation (image) is viewed differently by other professions and the public they serve. Consistent with previous studies, the PNs believe that formal uniforms, distinguishing devices and identity tags are important to earn the respect and trust of the parents/caregivers [51, 52].

Distinguishing devices indicating the qualification of the nurse, and name tags as adherence to patients’ rights, must be worn. The hair must be neat, the nails short, and the uniform must always be clean for infection-control purposes [1, 53]. This visual appearance makes it easy for parents/caregivers to identify the roles of staff members at the PHC facilities, reflects the PN’s preparedness to interact with them and their children, and enhances the PN’s professional appearance [54, 55].

Consistent with the findings of other studies, health care facilities require proper sanitation, staff and equipment, to limit cross-infection [56–58], as well as safe and welcoming spaces to cater for the children. The PNs were concerned about and demoralised by the broken toilets and unrestricted access to sharps containers, which were occupational hazards to staff and patients. Broken medical equipment also increased occupational hazards and caused longer waiting times [59, 60]. Training of staff on self-awareness, infection control and prevention policies, and accurate quality indicators may resolve or reduce the risk of infections and enhance safety [61, 62].

Shortages in recordkeeping documents, medication and vaccines jeopardised the EPI and continuity of care, aggravating the poor working conditions for the PNs and the inconvenience to parents/caregivers who must make follow up visits at the clinic, while the PNs must be reallocated to do catch-up campaigns for children who missed doses [63–65].

Limited space, overcrowding, and an environment that was not child-friendly concerned the PNs. Infrastructure problems in the PHC facilities, such as broken windows, tiles and toilets, were also challenging for the PNs. Overcrowding and poor ventilation pose a risk for transmissible diseases worldwide, SA included. During the pandemic, most resources were allocated to Covid-19, while screening and tracing of airborne diseases such as Tuberculosis (TB) were not prioritised, increasing the risk to the children visiting the PHC [56, 66]. Poor conditions in the working environment can be improved by deliberate human resource efforts for staff retention, accelerated equality, and communication among policymakers [67, 68].

### Limitations of the study

Data collection was time-consuming, due to the pandemic, which placed a heavy burden on PNs. The authors believe that this was the reason for the low response rate of (*n* = 8) participants from the (*N* = 25). The data-collection method, which were naïve sketches, yielded rich data, but allocating a month for the PNs to complete the naïve sketches at their own pace. Even though the researcher was available to assist by telephone or WhatsApp, this could have been the reason why some PNs signed the informed consent form but never participated; the process may have seemed long and lonely, since the researcher was not collecting data face-to-face, and all these factors led to a low response rate. Although the sample size was small, data saturation was reached after six naïve sketches. The findings of this study reflect only the participants’ perceptions and can therefore not be generalised, but can be used as a guide for other PNs in the practice environment.

#### Conclusion and relevance to clinical practice

Nursing professionalism is a concept with many definitions. The study departed from the RNAO 2007 [5] attributes in the nursing professionalism framework to explore the perceptions of PNs in PHC and hospital settings providing quality of care to neonates, infants and children under 5.

Seven of the eight original RNAO 2007 [5] professionalism attributes of nursing emerged; the exception was the attribute named innovation and visionary. However, a new attribute named ‘professional reputation’ emerged, representing both the nurse and the facility. Knowledge and acquisition are an evolving process that requires PNs to expose themselves to in-service training, to work according to the implemented clinical guidelines, and to strive for evidence-based research, in order to be a safe health care practitioner. Spirit of inquiry relates to lifelong learning, which can be informal, through short and refresher courses. A PN can follow a recognised formal training such as advanced courses, diploma or degree, and become a specialised practitioner with more skill and competency.

The PN must adhere to the scope of practice and acknowledge possible limitations that may cause unsafe practice or omissions. Accountability entails abiding by the health framework of SA’s Nursing Act 33 of 2005 and the safety of patients. Parents/caregivers have an autonomy role, opposite to the advocacy role of the PN. Both roles must be recognised, to improve the care of the child. The PNs in this study reckoned that for their own nurse autonomy to improve, there needs to be more visibility through campaigns, seminars and courses. The advocacy role requires cooperation from the parent/caregiver and the MDT, as well as keeping all records of all discussed health plans for the child. Collegiality and collaboration contribute to the partnership and acknowledge each other’s roles, and the PNs emphasise the interpersonal-relationship skills of good communication, mutual respect, and teamwork among colleagues.

The attribute of ethics and values for the parent/caregiver is trust and respect, which encourage adherence to treatment and follow-up visits for child health services. Challenges in health care may threaten safe and ethical practice, exposing the patient to harm and the PN to charges of malpractice. Even in such cases, incidents must be reported confidentially and privately in order to retain the child’s dignity. Ethical care or competency is accompanied by health education in a conducive environment for the parent/caregiver, according to the child’s needs.

Nurses’ reputation is viewed differently by other professions and the public they serve. PNs viewed themselves in terms of the external environment, qualifications and experience, and their MDT’s opinion [69–71]. Visual appearance, such as uniform and impeccable personal hygiene, represents PNs as committed and improves trusting relationships. Facility reputation is important for the safety of the child and the community by observing infection-control measures. The work space needs to be well-ventilated and welcoming.

PNs also need to be creative, critical thinkers who collaborate with other stakeholders; to be inspired and skilled for innovative and visionary skills in care; and need to know the new direction of future health care services [72–74]. Interest must be ignited in the PN, such as creating awareness of innovation projects, benchmarking, and open invitations for leadership training. However, in the South African context, professional reputation relating to the nurse and the health facility is highlighted. This is relevant to SA, as the nursing profession and health facilities have been negatively perceived by the public, with complaints of incivility towards patients, poor infrastructure, and lack of resources.

### Recommendations for practice

- PNs tasked with the care of neonates, infants and children under 5 work in a MDT to provide quality care. It is therefore important that professionalism must be promoted amongst all health care practitioners (HCPs).
- Active promotion strategies from the DoH and health care institutions must be implemented, such as an Awareness Day, frameworks, and promotional materials like posters, and creating health policies that promote professionalism among all HCPs.
- In-service training is also cost-effective and should be utilised to educate HCPs about professionalism attributes and to create awareness of the concept and its implications on quality care.
- PNs must be encouraged to attend research days so that they are aware of how new findings in research can positively change the practice environment to enhance professionalism attributes for quality care.
- Nurses’ Day celebration and imbizos are platforms where nursing professionalism attributes and outcomes in health care can be discussed and emphasised.

#### Recommendations for education

- Knowledge is the fundamental attribute of nursing professionalism.
- It is proven that nursing professionalism can be learned, and the PN must keep up to date with evidence-based data to provide quality care to neonates, infants and children under 5.
  A well-established curriculum at the Nursing Education Institutions must be presented, starting with the undergraduate programme, with a clear assessment plan to instil the importance of professionalism as a necessary skill to be acquired to meet quality care requirements in health care.

- The Nursing Education Institutions need to collaborate more with clinical health facilities to educate and train PNs already in practice through workshops and work-based outcome strategies that will not be time and resource consuming.

#### Recommendations for policy

- Policymaking is a national initiative, and all stakeholders must partake. It was evident that the parents/caregivers of neonates, infants and children under-5 are decision-makers in their health outcomes; therefore, they must be included.
- The health care environment has a great influence on nursing professionalism. Parents are part of the group that evaluates PNs’ professionalism, and they must be aware of the stance of the DoH and the SANC on professionalism, so that there is a clear expectation from all parties.
- Policy-making has been promoted as a task of management. Nurses from all levels and categories must be openly invited to be actively involved in policy-making, and be provided with opportunities such as a day off to travel.
- These policies must be easily accessible after approval, and they can be simplified into a poster with images, be discussed on media and/or be electronically shared with everyone who has a device to receive electronic documents.
- Continuous reviews are necessary as well, so that new information or strategies are incorporated to improve nursing professionalism and quality of care.

#### Recommendations for research

- Available research on nursing professionalism is insufficient to support quality care of the neonates, infants and children under 5.
- This can also be extended to another context and in a larger population, especially on the African continent where child mortality is high. It is also recommended that each attribute be explored individually, in order to fully understand each attribute.
- Based on the findings of this study, the attribute of innovation and visionary did not emerge. It is important to explore the cause of these attributes’ absence.
- A PN needs to find new ways of implementing quality care. PNs need to be encouraged more, early in their undergraduate and post graduate training, to undertake research.
- It was evident that PNs are aware of their perceptions of nursing professionalism to provide quality care to neonates, infants and children under 5. Therefore, it is recommended that further studies be undertaken to investigate or explore specific challenges that inhibit nursing professionalism in this context, as evidenced by the high mortality rates in sub-Saharan Africa.
- The concept of nursing professionalism is known in sub-Saharan Africa; this study has found that “professional identity” has similar attributes to professionalism, as published by the International Society for Professional Identity in Nursing. This concept could be an area of research interest, with particular focus on sub-Saharan Africa.

## Supplementary Information

Supplementary Material 1.

Supplementary Material 2.

Supplementary Material 3.

## Data Availability

The authors declare that all the data generated or analysed during this study are included in the supplementary files and the published article.

## Abbreviations

Covid-19: Coronavirus disease-19
DoH: Department of Health
EPI: Expanded Programme on Immunisation
HIV: Human Immunodeficiency Virus
HREC: Health Research Ethics Committee
IMCI: Integrated Management of Childhood Illnesses
MDT: Multi-disciplinary team
NWDoH: North West Department of Health
PHC: Primary Health Care
PNs: Professional Nurses
RNAO: Registered Nurses’ Association of Ontario
SA: South Africa
SANC: South African Nursing Council
SDGs: Sustainable Developmental Goal/s
TB: Tuberculosis
UN: United Nations

## Data availability

The authors declare that all the generated and analysed data during the study is provided within the manuscript and supplementary files.
