# Supplementary material for: Nurses’ perceptions regarding their own professionalism attributes to quality neonatal, infant and under-5 childcare
Source: BMC Nurs. 2024 Oct 8;23:727. doi: 10.1186/s12912-024-02375-0 (PMC11463118; doi:10.1186/s12912-024-02375-0)
Supplement: Supplementary file 1 — Supplementary Material 1. [file 12912_2024_2375_MOESM1_ESM.docx]

**Table S1: Criteria and strategies of trustworthiness and the application thereof**

| **Criteria for trustworthiness** | **Strategies for trustworthiness** | **Application of strategies** |
| --- | --- | --- |
| **Truth value:** implies that the researcher has established confidence in the truth of her data and findings in the context of where the study was conducted. | **Credibility:** the researcher was objective in the process of data collection and the results reflected the participants’ own perceptions. | Participants could contact the researcher at any time with questions during the naïve sketch process.  An independent co-coder was involved in data analysis.  The participants were verbally reminded during the PowerPoint presentation, on the information page and via WhatsApp to write legibly with a dark pen to ensure the accuracy of data analysis.  The participants were also requested to write their own perceptions without receiving help or input from anyone else.  The participants were contacted for clarity when the handwriting was not legible.  Data saturation was reached from the sixth of eight naïve sketches. |
| **Applicability:** is the degree to which the findings of a particular study can be applied in another setting and population. | **Transferability:** the researcher did not seek to generalise the findings of this study to a larger population, but a detailed description of the research methodology was provided for other researchers to follow. | The research design and methodology are clearly described to ensure the study can be repeated.  All-inclusive sampling enabled rich data that was received from the PNs who are knowledgeable in the care of neonates, infants and children under-5 in the North West Province and the need to decrease this population’s mortality rate.  A thick description of the research design and methodology is given to enable transferability. |

**Table S1: Criteria and strategies of trustworthiness and the application thereof – continued**

| **Criteria for trustworthiness** | **Strategies for trustworthiness** | **Application of strategies** |
| --- | --- | --- |
| **Consistency:** to consider whether another study would yield the same results if repeated on the same population. | **Dependability:** the researcher provided the research design and methodology for the study to be repeated. | To ensure consistency, the research design and methodology were thoroughly described to enable other researchers to duplicate the study, and the process was precisely applied throughout the research. |
| **Neutrality:** the research needs to refrain from bias throughout the research process. | **Confirmability:** the researcher remained objective in the analysis process, and only data provided by the participants was used. | An independent co-coder was involved in data analysis to ensure non-bias, as well as the supervisors as experts in the field of research.  A thorough literature review was conducted to support the findings. |

Source: (Guba & Lincoln)
