# Supplementary material for: Nurses’ perceptions regarding their own professionalism attributes to quality neonatal, infant and under-5 childcare
Source: BMC Nurs. 2024 Oct 8;23:727. doi: 10.1186/s12912-024-02375-0 (PMC11463118; doi:10.1186/s12912-024-02375-0)
Supplement: Supplementary file 2 — Supplementary Material 2. [file 12912_2024_2375_MOESM2_ESM.docx]

| **Table S2: Demographic characteristics of participants** | | | | | | | |
| --- | --- | --- | --- | --- | --- | --- | --- |
| **Age** | **18-29** | **30-35** | **36-40** | **41-45** | **46-50** | **51-60** | **61+** |
|  | 4(50%) | 1(12.5%) | 2(25%) | 0(0%) | 1(12.5%) | 0(0%) | 0(0%) |
| **Gender** | **Male** | **Female** |  |  |  |  |  |
|  | 0(0%) | 8(100%) |  |  |  |  |  |
| **Marital status** | **Single** | **Married** | **Widowed** | **Divorced** | **In a relationship** | **Other** |  |
|  | 3(37.5%) | 3(37.5%) | 0(0%) | 0(0%) | 2(25%) | 0(0%) |  |
| **Number of children** | **0** | **1** | **2** | **3** | **4** | **5+** |  |
|  | 2(25%) | 5(62.5%) | 0(0%) | 1(12.5%) | 0(0%) | 0(0%) |  |
| **Home language** | **Setswana** | **Sesotho** | **Afrikaans** | **Ndebele** | **Zulu** | **Other** |  |
|  | 4(50%) | 1(12.5%) | 1(12.5%) | 1(12.5%) | 1(12.5%) | 0(0%) |  |
| **Qualification** | **Degree** | **Diploma** |  |  |  |  |  |
|  | 3(37.5%) | 5(62.5%) |  |  |  |  |  |
| **Work experience (years)** | **1-5** | **6-10** | **11-15** | **16-20** | **21-25** | **26-30** | **30+** |
|  | 5(62.5%) | 0(0%) | 2(25%) | 1(12.5%) | 0(0%) | 0(0%) | 0(0%) |
