# Supplementary material for: Nurses’ perceptions regarding their own professionalism attributes to quality neonatal, infant and under-5 childcare
Source: BMC Nurs. 2024 Oct 8;23:727. doi: 10.1186/s12912-024-02375-0 (PMC11463118; doi:10.1186/s12912-024-02375-0)
Supplement: Supplementary file 3 — Supplementary Material 3. [file 12912_2024_2375_MOESM3_ESM.docx]

**Table S3: Perceptions of PNs regarding their own professionalism attributes to provide quality care to neonates, infants and under-5s**

| **Categories** | **Themes** | **Sub-themes** | **PNs “Verbatim” quotes/responses** |
| --- | --- | --- | --- |
| Knowledge | Knowledge acquisition | In-service training | It is also helping now, and then we are being sent for a refresher course and doing in-service training *(1/8/F/36-40).*  Paediatricians give in-service training to PNs and other nursing categories in the paediatric unit about management and caring for neonates, babies and under-5 children *(7/8/F/46-50).* |
|  |  | New guidelines | Guidelines such as HIV [Human Immunodeficiency Virus], TB [Tuberculosis], EPI [The Expanded Programme on Immunisation] and IMCI [Integrated Management of Childhood Illnesses] also guide to maintain the standard of continuation of the skills and knowledge *(3/8/F/36-40).*  Guidelines that will help in providing quality care to neonates, babies and under-5 children, such guidelines should be revised annually for changes and additions *(7/8/F/46-50).* |
|  |  | Evidence-based practice | When there are newly updated guidelines, the nurse who works with children goes for training and does in-service training as feedback to other nurses *(5/8/F/30-35).*  I am responsible for keeping up with new research and training; by doing that, the quality of care I provide to neonates, infants, and under-5 child is exceptional or the best that I can provide *(2/8/F/18-29).*  In the nursing field, there are continuous changes and new evidence-based practice that leads us on how to provide care constantly *(2/8/F/18-29).* |
| Spirit of inquiry | Lifelong learning | Short courses | It’s going to short courses like refresher courses to remind you how things are done, like IMCI *(1/8/F/36-40).*  To advocate for my patient and deliver quality nursing care, I need to go on training and do short courses that would help in giving proper services to the clients *(6/8/F/18-29).* |
|  |  | Advanced courses, diplomas, and degrees | I also developed myself by studying Advanced Management and Advanced PHC [advanced courses], and that development helped to gain more knowledge to maintain good quality in neonates, infants, and children under 5 *(3/8/F/36-40).*  The more nurses take responsibility for professional development by further studying, e.g., Paediatric speciality [diplomas and degrees], the more the chances of providing quality services to infants, neonates and under-5 years children *(5/5/F/30-35).*  Having a nurse with specialised qualifications [advanced courses] helps in saving lives and improves the quality of care provided for our young ones *(7/8/F/46-50).* |
| Accountability | Responsibility for good practice | Scope of practice, acts, omissions, mismanagement | Should you perform duties that are against your scope of practice and is not in line with the departmental policies and protocol, you will be summoned for a disciplinary hearing, and you will have a bad reputation [acts and omissions] *(7/8/F/46-50).*  IMCI and EPI – this helps a lot for me to render care for these babies and children and improve my skills and responsibility/accountability as a nurse [scope of practice] *(4/8/F/18-29).*  Taking responsibility for your actions. Learning from your own mistakes [acts and omissions] *(8/8/F/18-29).*  Litigations by parents whereby the staff/personnel is sued for mismanaging their child *(7/8/F/46-50).* |
| Autonomy | Enhancing career independence | Campaigns, seminars and courses | Promotes the nursing profession to communities to reach more people *(2/8/18-29).*  Increasing your awareness about your professionalism *(2/8/18-29)*  Organising seminars for PNs and paediatricians in caring for neonates, babies and children under 5 *(7/8/46-50).*  Campaigns are to be conducted quarterly regarding the health of neonates, babies and under-5 children |
| Advocacy | Communication between multi-disciplinary team members (MDT) | Recordkeeping | To record clearly and logically, using neat and correct handwriting *(3/8/F/36-40).*  Importance of treatment and return dates and immunisation coverage *(3/8/F/36-40).* |
|  |  | Referrals | Advocacy: as a nurse, I have all the right to advocate for these children, especially where I see/notice that the child is being abused or neglected, like involving, for example, a social worker [referral] if there is a need *(4/8/F/ 18-29).*  PHC facilities attend patients at a primary level of health care within the community and only refer to secondary or tertiary level of health care when there is a complication *(5/8/F/30-35).*  Being able to advocate for my patients when something is not done. For example, in a case where the doctor refuses to refer a child that needs a referral. It is the nurse’s responsibility to advocate for the child as this can improve your quality of nursing or the health of that child need(ing) a referral *(8/8/F/18-29).* |
| Collegiality and collaboration | Collegial interrelationships | Mutual respect and trust | Respect other co-workers’ cultures. The PNs must be a trustworthy person and always be fair, and just applies rules as needed; as a result, it becomes easier and more comfortable working as a team and achieving set objectives *(3/8/F/36-40)*.  Respect is one of the important factors, respecting clients and colleagues, including other people’s opinions and decisions. *(7/8/F/46-50).*  Trusting each other as colleagues also makes the work easy, and colleagues won’t be afraid to help you when you ask for their help *(8/8/18-29)* |
|  |  | Good collegial communication skills | Child care includes the following: good communication and healthy relationships. It is important to have good interpersonal relationships with colleagues, as with parents of the patients *(2/F/18-29).*  Communication with other members of the team, i.e., doctors, dieticians, social workers etc., by sharing important information which will help improve the health of our clients and help offer quality care *(7/8/F/46-50).* |
|  |  | Teamwork and team spirit | MDT members work together [teamwork] to improve the quality of care of the neonates, infants and under-5 children *(3/8/F/36-40).*  Other health workers need to be included, such as social workers, doctors, health promoters, dietitians and mother mentors *(6/8/F/18-29).*  The good relations [team spirit] amongst colleagues help(s) contribute to the quality of care of children since they work together as a team to help children from birth until five years *(5/8/F/30-35).*  I need the help of a cleaner or general worker to keep the place clean *(6/8/F/18-29).*  Being able to work together as a team *(8/8/F/18-29).* |
| Ethics and values | Towards parents/caregivers | Form trust and respect relationships | Personality is welcoming enough for the parent of the infant or neonate; they would be comfortable enough to trust me to tell me all the history needed to make a proper diagnosis *(6/8/F18-29).*  It means being more flexible towards the parent because the patients become more trustworthy towards the nurse…be more friendly…when communicating well with the clients they listen well *(1/8/F/36-40.)*  To establish trust amongst patients, present well with happy faces/smiles. On uniform identified with distinguishing devices and name tags so that patients can respect, trust, and know their names and the work they do *(5/8/F/30-35)*  Being honest to children and their parents, establishing boundaries, being an active listener and always showing respect *(5/8/F/30-35).*  Always maintain privacy and confidentiality [with] regard to the patient’s health/diseases. Prioritising nursing ethics and values about humanity and knowledge of clinical practice, which involves individual moral values *(3/8/F/36-40).* |
|  |  | Confidentiality and privacy | Patients’ information, especially of children, should be safeguarded, which means their record files should be kept in a safe area where no unauthorised person can get hold of them. Privacy – just because these are children doesn’t mean their right to privacy must be violated. This means, for example, when assessing a child, privacy has to be maintained like screening curtains when undressing the child and keeping their dignity *(4/8/F/18-29).* |
|  |  | Good communication skills | There must be an interpersonal relationship between us health workers and the carer, and the nurse. It is important because the carer is the one communicating with me on behalf of the child *(4/8F/18-29).*  Child care includes… good communication and healthy relations. It is important to have good interpersonal relationships with work colleagues as well as the parents of the patients *(2/8/F/18-29).*  The PNs must have listening skills with the parents of the neonates, infants and under-5 children in order to collect correct history and information *(3/8F/36-40).*  Listening skills [communication skills], be able to listen to the parents/guardian’s concerns regarding the health/wellbeing of their babies *(7/8/F/46-50).* |
|  |  | Health education | When communicating well with the clients, they listen well, especially when giving health education… they can be able to use that information at home and help others also the community *(1/8/F/36-50).*  Giving clear information through good, effective communication to these parents. This will empower them with knowledge regarding their kids *(2/8/F/18-29)*.  Giving health education to encourage mothers that is important to bring their babies to the clinic *(3/8/F/36-40).* |
| Professional reputation | Professional nurse | Clean uniform, distinguishing devices name tags | It is like wearing the correct uniform and our professional [distinguishing] device; sometimes the mothers are trusting who are wearing the correct uniform or devices like epaulettes to treat their children *(1/8/F/36-40).*  If I am neat in my uniform, wearing my distinguishing devices and name tag, the parents of their children respect me and are more likely to trust me to take care of their children *(2/8/F18-29).*  The PNs and the MDT members must look presented daily, e.g., they must be in proper uniform, have name tags and their appearance [presentable], long nails are avoided, and long hair must be fastened to look tidy and avoid cross infection *(3/8/F/36-40).*  To establish trust amongst patients, present well with happy faces/smiles on uniform identified with distinguishing devices and name tags so that patients can respect, trust and know their names and the work they do *(5/8/F/30-35).*  Previously the nursing managers or matrons were strict when coming to professional appearance, i.e., uniform must be clean *(7/8/F/46-50)*.  As a nurse, it is always important to always be able and professional. For example, being neat and always in your uniform shows how much you value your work and those you are working with, and you will be [good at] nursing *(8/8//F/18-29).* |
|  |  | Personal hygiene (hair and nails) | Applying make-up, like long nails or nail polish sometimes can scratch the baby or be more dangerous, or sometimes the long nails can hold infections underneath and cause infections in children *(1/8/F/36-40).*  Long nails must be avoided. Long hair must be fastened to look tidy and avoid cross-infection *(3/8/F/36-40).*  No long hair, no long nails, no wearing of nail polish, and no jewellery because it can cause harm to patients *(7/8/F/46-50).* |
|  | Health care facility | Infection control | The cleanliness of the facility, as some children are crawling, so if it is dirty on the floor, children can get infections, it is important to clean *(1/8/F/36-40).*  Healthcare can’t be practised in a dirty environment. The facility must be clean, as well as the consulting room *(2/8/F/18-29).*  Infection-control measures are to be taken at the facility to prevent cross infections to neonates, infants, and the child under 5, e.g., washing hands every [time] after touching patients. *(3/8/F36-40).*  Poor ventilation in the facility can expose infants to infections and conditions like TB and Covid-19. Children and toddlers like to walk and fiddle with objects, so if they are not cleaned, or the facility is not safe enough for them, they stand a high chance of injuries and contracting infections *(6/8/F/18-29).*  It is very important to work in a clean environment with no infections. Infection-control measures to be in place to avoid the spread of infections and make sure that children’s health won’t be affected *(8/8/F/18-29).* |
|  |  | Safety and needle disposal bins | In a safe environment, for example, needles are secured in a safe container after use, not in the bed, which prevents injuries *(2/8F/18-29).*  Syringes and needles should be discarded safely in the sharps container and kept away from the reach of children. Children tend to play with anything they come across; at times, they put those things in their mouths *(7/8/F/46-50).* |
|  |  | Adequate equipment, medication, and nursing staff | Correct equipment to treat or diagnose the patient, like thermometers, are not available, you won’t be able to diagnose the children, or if you don’t have scales to weigh the babies, you won’t be able to diagnose malnutrition *(1/8/F36-40).*  Availability of resources like equipment to carry out procedures, patients’ folders for recordkeeping for future reference, enough linen, and beds to avoid babies sharing beds that might lead to cross-infection of diseases *(7/8/F/46-50)*.  Availability of medication and medication cupboard, which is lockable. Availability of sterile dressing packs to perform daily dressings that will prevent wounds from being septic *(7/8/F/46-50).*  Shortage of staff can also contribute because you won’t be able to do quality work if you are pushing to finish the line *(1/8/F/36-40)*.  The availability of staff and working shifts will reduce burnout syndrome and fatigue *(7/8/F/46-50).*  This can also include the availability of staff because for us to render quality nursing care, each one of us must do their duties *(6/8/F/18-29).* |
|  |  | Available, pleasant atmosphere in consultation rooms | The facility must always be welcoming and have all the resources that are needed to deliver the services because if there are no resources, it becomes difficult to deliver good service *(8/8/F/18-29).*  It also must be tidy and organised *(2/8/F/18-29).* |

The coding at the end of each excerpt is a formula; for example “(1/8/F/36-40)”, 1/8 identifies the naïve sketch number, F refers to female, and 36-40 refers to the participant’s age group.
